# Impact of Protracted Displacement on Delay in the Diagnosis Associated with Treatment Outcomes: A Cross-Sectional Study in Internally Displaced Tuberculosis Patients of Pakistan

**DOI:** 10.3390/ijerph182211984

**Published:** 2021-11-15

**Authors:** Farman Ullah Khan, Faiz Ullah Khan, Khezar Hayat, Jie Chang, Muhammad Kamran, Asad Khan, Usman Rashid Malik, Asif Khan, Yu Fang

**Affiliations:** 1 Department of Pharmacy Administration and Clinical Pharmacy, Xi’an Jiaotong University, Xi’an 710061, China; farman.khan@abasynisb.edu.pk (F.U.K.); fkhan@bs.qau.edu.pk (F.U.K.); khezar.hayat@uvas.edu.pk (K.H.); jiechang@xjtu.edu.cn (J.C.); usmanmalik_ucp@hotmail.com (U.R.M.); 2 Center for Drug Safety and Policy Research, Xi’an Jiaotong University, Xi’an 710061, China; 3 Shaanxi Center for Health Reform and Development Research, Xi’an Jiaotong University, Xi’an 710061, China; 4 Research Institute for Drug Safety and Monitoring, Institute of Pharmaceutical Science and Technology, Western China Science & Technology Innovation Harbor, Xi’an 710061, China; 5 Institute of Pharmaceutical Sciences, University of Veterinary and Animal Sciences, Lahore 54000, Pakistan; 6 Department of Pharmacy, Faculty of Biological Sciences, Quaid-i-Azam University, Islamabad 45320, Pakistan; kamifidipharmd@gmail.com (M.K.); assad.pharmacist@gmail.com (A.K.); 7 District Bannu TB Control Program Unit, Headquarter Hospital Bannu, Bannu 28100, Pakistan; asiftb001@gmail.com

**Keywords:** tuberculosis, delay in diagnosis, treatment outcomes, internally displaced TB patients

## Abstract

Human displacement is on the rise globally, and the increase in the burden of tuberculosis (TB) is also attributed to migrations worldwide. A significant number of such displacements occur in regions with considerably higher areas of TB burden. Displacements may delay TB diagnosis and treatment, which will possibly lead to TB transmission among healthy individuals. In this study, we assessed the association of existing determinants after a protracted internal displacement of people with delay in TB diagnosis and treatment outcomes. A cross-sectional study was conducted on internally displaced TB patients (IDPs), registered at selected health facilities in three urban districts of Pakistan from March 2019 to February 2020. The univariate and multivariate logistic regression model was used to assess the delay in diagnosis and treatment outcomes. IDPs with delay in initiation of treatment beyond 30 days were at high possibility of unsuccessful TB treatment outcomes (adjusted odds ratio AOR, 2.60; 95% CI 1.06–6.40). Furthermore, the multivariate regression analysis showed a statistically significant association (*p* > 0.05) between TB patients who were aged 55 to 65 years (AOR, 2.66; 95% CI 1.00–7.07), female patients (AOR, 2.42; 95% CI 1.21–4.81), visited non-formal health provider (AOR, 8.81; 95% CI 3.99–19.46), self-medication (AOR, 2.72; 95 % CI 1.37–5.37), poor knowledge of TB (AOR, 11.39; 95% CI 3.31–39.1), and perceived stigma (AOR, 8.81; 95% CI 3.99–19.4). Prolonged delay in treatment was associated with unfavorable treatment outcomes among IDPs. Migrants and IDPs are more likely to experience an interruption in care due to overall exclusion from social and health care services. Therefore, it is imperative to understand the barriers to providing public health care services, particularly in preventing and treating TB.

## 1. Introduction

Tuberculosis (TB) is one of the leading causes of public health problems in developing countries [1]. According to the WHO 2020 TB report, about 7.1 million TB cases were reported. Only eight developing countries accounted for two-thirds of global TB cases. Pakistan was placed fifth among them [2]. The National TB Control Program of Pakistan (NTP) reported that the number of TB cases annually was 630,000 (364/100,000 people), with a death rate of 34/100,000 people [3]. The WHO initiated End TB Strategy sets 2035 targets to achieve two goals, i.e., up to 90% annual reduction in the incidence rate, and 95% reduction in TB deaths [4]. Although developments are being made, the progress has remained very slow in developing countries, and it is predicted that the world will not see an end to TB by 2035 as anticipated [5]. To achieve these goals, the WHO emphasize avoiding delays in the diagnosis and treatment of TB. Previous studies reported that the main barrier to TB control is undetected and untreated TB patients [6]. Delay in diagnosis and treatment will not only increase the risk of disease severity but also promote its transmission in healthy individuals [7]. A single active tuberculosis patient is estimated to transmit the illness to around 10–15 healthy individuals [8]. Therefore, it is necessary to take major steps to address the challenge of undetected people with TB at the national and subnational level in low-and middle-income countries (LMICs), especially among the most vulnerable TB populations. One of the main barriers in high TB burden countries is passive case detection of TB [9]. In passive case finding, people with probable TB come to a public health facility and are investigated for TB and the initiative comes from patients [10]. Due to this passive case detection, many factors contribute to the delay in the diagnosis and treatment of TB, including poor socioeconomic status, awareness of symptoms of TB, living in poor hygiene conditions, social stigma, disease severity, access to health facilities, and situations of displacement emergency [11]. Another challenge to TB control is that multiple socioeconomic, cultural, environmental and political factors underpin the recent increase in human migration in developing nations [12]. These armed conflicts and population displacements have negatively contributed to the changes in patterns of TB infection and have an alarming effect on its overall management [13]. Several crisis-related changes, such as living conditions, irregular residency, illiteracy, malnutrition, overcrowding, social isolation, and disruption to health services, are reasons to be considered as risk factors for TB [14,15]. The total number of people living in conditions of internal displacement reached a record 48 million by the end of 2020 [16]. In Pakistan, due to conflict and violence in 2014, a large number of residents of the former federally administered tribal area of North Waziristan (NW) were forced to move to adjoining areas of Khyber PakhtunKhwa such as Lakki Marwat, Bannu, Frontier Region of Bannu, and Dera Ismail Khan. The United Nation Office has registered 961,000 IDPs from NW [17], who were forced to live with compromised living conditions and medical facilities [18]. Previous studies in Africa reported that armed conflicts had shown a negative impact on TB control programs due to interference with the goals of TB diagnosis and treatment [19]. In Ethiopia, TB patients from war-influenced regions delayed twice as long to seek treatment [20]. Similarly, in Syria, forced mass displacement and humanitarian law violations have hampered the ability to diagnose, treat, and follow up TB cases [21]. The displaced population faces several new challenges in avoiding TB risks and initiating proper treatment after displacement. Therefore, TB care demands a logical reaction to identify all the barriers to TB management. There is limited evidence on the influence of delay in diagnosis or treatment outcomes in internally displaced TB patients. Therefore, this study measured the association of existing determinants after prolonged internal displacement with delay in diagnosis and treatment outcomes.

## 2. Materials and Methods

### 2.1. Study Design 

**Figure 1:**
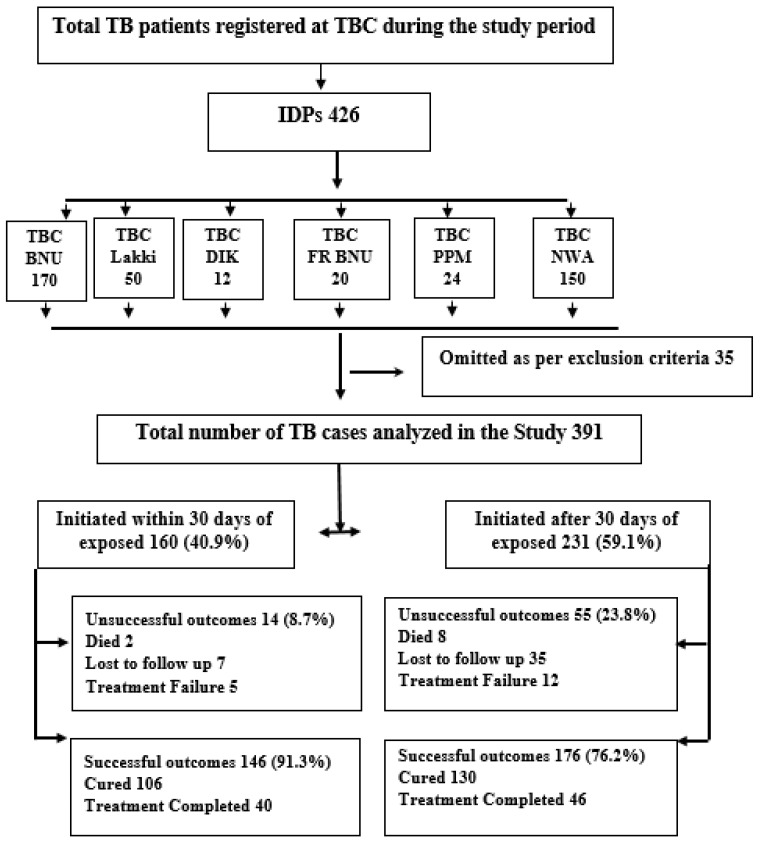
TBC (Tuberculosis Control Center), IDPs (Internally displaced TB patients), NW (North Waziristan), BNU (Bannu), F.R BNU (Frontier Region Bannu), DIK (Dera Ismail Khan), Treatment outcomes successful (cured and treatment completed), Unsuccessful treatment outcomes (treatment failure, treatment defaulter, died), Omitted participants (transferred out or registered patients whose outcomes are not known after completing the course).

A cross-sectional study was conducted in four selected TB control centers to examine risk factors related to the delay in receiving diagnostic and treatment initiation among internally displaced TB patients (IDPs) registered in TB centers in the Khyber Pakhtunkhwa (KPK) province of Pakistan (Figure 1).

### 2.2. Study Population and Study Setting

TB patients registered in any of the selected TB centers (TBC), which were located in public hospitals of Bannu, Dera Ismail Khan, the former Frontier Region of Bannu, and Lakki Marwat and to whom the treatment schedule was to be completed during the study period (March 2019 to February 2020), were enrolled in this study. Those TB patients who (a) refused to consent/participate, (b) were younger than 18 years and older than 65 years of age, and (c) could not recall the vital information required for the study were excluded from the final analysis. In order to avoid cross-contamination with local TB patients, the principal investigator reconfirmed the status of IDP patients during personal contact. 

### 2.3. Study Tool 

Information from patients with tuberculosis was collected on a predesigned questionnaire, which had three subcategories of variable, namely sociodemographic factors (e.g., residency, gender, age, education, and socioeconomic status), clinical factors (e.g., history of symptoms, category of treatment, treatment outcomes and the number of patients with tuberculosis in the family member’s dwelling) and health-related factors (e.g., delay in diagnosis, health-seeking behaviors, and distance from the health care center).

During patients’ visits to the TBC, information about socio-demographics, displacement, and health-related factors were recorded on a questionnaire after obtaining consent. Information about the patient’s diagnosis, treatment type, treatment start date, and treatment outcomes were taken from the TB01 form, TB02 card, and TB03 register of TBC. The TB01 form is the main source of information, filled out for every newly diagnosed patient, and bears important administrative and treatment-related information. The copy of the information of TB01 is recorded in the registers (TB03) maintained at TBC and used to keep track of the entire TB patient population in the area. TB02 is the TB patient’s card, kept with patients, containing general information about the patient and specific medical information about disease and treatment [22,23]. 

### 2.4. Operational Definitions

According to WHO and NTP guidelines, treatment outcomes were classified as successful when the TB patient completed the treatment medication and/or was ‘cured,’ while unsuccessful treatment outcomes were classified as treatment failure, treatment defaulter, died, transferred out, or registered patients whose outcomes are not known after completing the course [22,24]. 

Based on start of TB treatment after the onset of the symptoms, patients were classified into “Delayed” and “Not-Delayed,” taking 30 days (4 weeks) as cut-off points [25,26]. Diagnostic delay was taken as the time interval between the onset of the symptoms (major symptoms: cough, expectoration, and common symptoms: chest pain, mild fever, night sweats, poor appetite, and weight loss) and the date of labeling as TB patient after medical consultation. Duration to initial health-seeking was defined as the interim between the period of self-reported onset symptoms and the date of the first appearance at any health care facility (including hospital, TB dispensary, TB health center, etc.). The initial healthcare-seeking was said to be delayed if they visited the TB health-facility more than 3 weeks after the onset of symptoms [27]. 

All the cases were followed until the earliest treatment outcome. A new case of pulmonary TB was defined as a patient who had been registered for less than 2 months before the start of the study or who was registered during the study period (received category I treatment), while category II patients are previously treated cases who are receiving treatment after loss to follow up, relapse or failure. Both categories I and II receive first line of drugs; the difference is in the addition of streptomycin and duration of treatment for category II patients [28,29]. 

After the appearance of symptoms, patients who sought self-medication (e.g., cough syrups, antipyretics, and antibiotics available over the counter) instead of visiting a health facility were considered as self-treatment. A household contact was defined as a group of people who lived together, shared the same housekeeping arrangements, and ate together within one residence for at least 30 days prior to the identification of TB [30].

### 2.5. Data Analysis 

The sample size was calculated by using Rao soft calculator^®^ (Rao soft, Inc, Seattle, WA, USA), using a 95% significance level, and keeping the treatment adherence rate at 80% and 5% precision of the estimate. For data analysis, Statistical Package for the Social Sciences SPSS^®^ (IBM Chicago, IL, USA, version 23), was used. Data were presented with percentages and frequencies for the socio-demographic treatment delay and outcomes. To assess the delay >30 and ≤30 days and treatment outcomes among the participants, the univariate and multivariate logistic regression models were used. For variables that were significant in the univariate analysis, we measured a stringent univariate (*p* < 0.15), for addition of variables in the multivariate analysis model. All factors measured in univariate analysis were established with literature assessment and clinical importance of TB. In developing the multivariate binary logistic regression, we have checked the collinearity and tolerance value for all these variables. The presence of significant intercorrelations between two or more independent variables in a regression model is known to as multicollinearity. In case of independent variables having high correlation with each other (Variance inflation factor = 10 and Tolerance value > 0.1), one of them was taken out from the final analysis model. The Hosmere Lemeshow test was also applied for the standardization of the final logistic regression model [31,32]. Two different categories with binary variables were made for treatment outcome, i.e., successful and unsuccessful and delayed and not delayed. Odds ratios with 95% confidence intervals *p*-value < 0.05 were considered significant and statistically important.

## 3. Results

### 3.1. Characteristics of Participants 

A total of 391 TB patients were enrolled in the study. Of the total, 49.6% were from the rural area, 35.3% from the camps, and 15.5% were from the city area. The majority of the TB patients were male (52.2%) with a median age of 18–25 years (27.4%). The vast majority of respondents (334, 85.4%) were illiterate. Most of the reported (134, 34.3%) patients have to travel more than 30 km to reach the health center. The majority of the participants also faced living in an overcrowded situation (166, 42.5%) Table 1. 

### 3.2. Health-Seeking Attitude 

More than half of the IDPs (59.1%) obtained treatment after more than 30 days of delay since they first experienced TB symptoms. Most of these patients (391, 91.7%), started TB treatment in TB diagnosis centers where they had been diagnosed (Figure 1). Most of the IDPs (286, 73.1 %), had first visited the non-formal health facility and used different self-medication (37.6%), before visiting a formal health care provider after the onset of initial TB symptoms. Overall 342 (87.5%), of the participants had inadequate knowledge of TB diagnosis, and only 12.5% were found to be knowledgeable, and 71.4% of IDP TB patients were identified with stigma related TB (Table 1).

### 3.3. Baseline TB Signs and Symptoms and Transmission amoung Patients 

When patients with a delay of more than 30 days were compared to those with a shorter delay, 381 patients (97.4%), reported persistent cough and statistically significant higher rates of fever, chest pain, and loss of body weight were identified (Table 2). Seventy-four (74) participants were identified as having more than one TB patient per house after the screening, which was the most likely probability of delay (AOR, 1.94; 95%CI 0.78–4.79) (Table 3). 

### 3.4. Predictors of Delay 

Based on the analysis, 59.1% of TB IDPs patients have shown a delay of >30 days (Figure 1). Consequently, multiple linear regression resulted in a statistically significant difference in terms of patients’ age considering 18–25 age group as a reference, 46–55 years odds ratio (AOR, 3.37; 95% CI 1.21–9.37), and 56–65 years odds ratio (AOR, 2.66; 95% CI 1.00–7.07), showing that both geriatric groups had a greater delay of more than 30 days in initiating TB diagnosis. Female patients have 2.42 times higher odds of probability of diagnostic delay as compared to males. There was also a statistically significant difference in delay amongst patients, with those who travelled more than 30 km indicating a high probability of delay in diagnosis with a 4.13 odds ratio (95 % CI 1.02–16.6), compared to those patients who traveled less than 30 km. Patients who first visited non-formal health caregivers are more likely to delay getting proper treatment and diagnosis (AOR, 8.81; 95% CI 3.99–19.46). Those who first practiced self-treatment with various therapies at their home were more likely, with an odds ratio (AOR, 2.72; 95 % CI 1.37–5.37), to experience longer delay. Additionally, poor knowledge of TB diagnosis before starting the treatment was also more likely to show delay in health care seeking (AOR, 11.39; 95% CI 3.31–39.1). Stigmatized patients were more likely to delay TB diagnosis and experience longer delay (AOR, 8.81; 95% CI 3.99–19.4) Table 3.

### 3.5. Predictors of Tuberculosis Treatment Outcomes

Overall, 82.4% of patients had a favorable treatment result (cured and treatment completed). A total of 69 (17.6%) patients had unsuccessful outcomes (treatment failure, loss to follow up, and death). The percentage of unsuccessful outcomes was more among patients with delay >30 days (55, 23.8%) (Figure 1). Those patients with a delay of more than 30 days were at risk of unsuccessful outcomes compared to those with a delay of < or equal to 30 (AOR, 2.60; 95% CI 1.06–6.40). A multivariate analysis also determined patients with geriatric age group (AOR, 3.00; 95% CI, 1.25–7.19), and those who have category of treatment II (AOR, 4.80; 95% CI 1.99–8.34) were significantly associated with unsuccessful treatment outcomes (Table 4).

## 4. Discussion

In this study, we identified a relationship between total delay and unsuccessful treatment outcomes. The rate of successful treatment outcomes was lowered from the WHO global target rate of 85% [33]. Most of the participants with a prolonged delay of >30 days were found to have unsuccessful treatment outcomes compared to participants with a delay of ≤30 days. However, delay in initiation of anti-TB medication results in various serious complications, which may significantly affect the treatment outcomes [34]. Unsuccessful treatment outcomes are associated with drug resistance against anti-TB drugs, which may enhance morbidity, mortality, disease transmission and treatment failure rate [35]. The results of this study underscore the need for rapid identification and management of tuberculosis, especially in a highly vulnerable population such as IDPs who were exposed to prolonged delays of >30 days [36]. The analysis showed a statistically significant association between prolonged treatment delay and unsuccessful treatment outcomes which is inconsistent with the findings of earlier studies [7,37,38]. This suggests the need for early finding of TB cases to ensure better results among IDPs and lessen the TB burden. The prolonged delay in diagnosis and treatment worsens the disease progression and symptoms [39]. Following the onset of symptoms, reporting first to the TB center was measured as a reference point in this study, an approach reported in 39 previous studies [40]. We found that the prolonged delay of >30 days was more common among IDP TB patients, in accordance with previous studies where the median delay of patients was 4 weeks and 6 weeks, respectively [9,41,42,43] and lower than studies conducted in Ethiopia, India and Chad [44,45,46]. The disagreement between these studies may be due to TB patients from different countries and their health systems, policies and infrastructure, and differences in sociodemographic characteristics. Health care-seeking behavior is a complex process influenced by many external and internal factors. IDPs showed a prolonged delay in seeking care because 73.1% of TB patients lean towards less expensive nonformal health services and 37.6% practiced self-medication, both leading to poor treatment outcomes. This is in line with the finding of previous studies conducted in conflict and non-conflict regions that non-formal healthcare providers play a significant role in delay [36,47,48]. Furthermore, a significant association was observed between self-medication and delay, which is consistent with previous studies [49,50]. Furthermore, distance from the TB center of more than 30 km is responsible for an unacceptable delay in patient care. This is supported by a considerable number of studies, systematic reviews and meta-analyses which specified that patients who travel a long distance to reach healthcare services experienced longer delays [51,52,53]. 

Our findings propose that coordinated efforts are needed to increase public awareness of the importance of the right diagnosis at the right time and seeking health in the right formal health care centers. In this study, due to lack of public awareness, the majority of the IDPs had no defined knowledge of TB treatment and diagnosis and have been living in a single shared room or in camps that have significantly affected patients reporting, diagnosis and timely initiation of treatment. The findings of the current study are in agreement with previous studies published elsewhere [29,41,54,55]. Our finding supported that lack of TB knowledge may lead to a patient unwillingness to seek suitable health care. 

Our study also observed that patients over 55 years of age and women have an increased probability of prolonged delay. Older patients and women in this part of the world generally depend on others, so it can be challenging for them to seek medical care when necessary. However, similar to our study, a systematic review of 45 studies from Asia stated that older age and females are strongly associated with delays in the diagnosis of TB [11,56,57]. Females’ tendency to delay has been attributed to their restricted socio-cultural environment in Pakistan, decision-making power, the burden of domestic work, illiteracy, and distance from health care centres [58]. 

The WHO reported that the most important determinant of TB diagnosis and treatment delay is stigma [59]. We found that prolonged patient delay was common in those who reported any form of stigma. Our findings are similar to those for other countries; studies observed stigma as a common obstacle in seeking early treatment [60,61,62]. Available evidence shows that interventions are needed to reduce TB stigma, and there is a need for patient-centered TB care [29].

The delay in diagnosing TB patients is also related to the increased spread of the disease in healthy individuals [36,44]. Time spent in crowded camps increases the risk of TB exposure and infection. Timely identification is very important for TB control because delayed diagnosis is known to be linked with higher TB transmission rates [36]. This may be one of the reasons why the participants from IDPs accounted for more TB cases and experienced a higher rate of TB cases in their families. The result of our study is similar in this context to other studies [36,63]. 

The most prominent symptoms associated with the development of TB were the appearance of a persistent cough. However, a significant percentage of TB patients were experiencing symptoms such as fever, chest pain, and weight loss, particularly in patients whose diagnosis and treatment were delayed for more than 30 days. The result of this study is in agreement with previous studies [7,64]. 

This study has several limitations. First, the reporting interval of symptoms is based on the patient’s recall and interpretation, and recall bias is a serious issue for an accurate estimate of delay. Unfortunately, there is no feasible method to deal with the issue of recall bias except to include recently treated patients. The response of the inaccessible TB patients, who met the inclusion criteria, could not be analyzed. Hence, the outcomes cannot be summed up to all TB patients in other parts of the country.

The study has several pros and cons. As far as the significance of the study is concerned, this is the main study in Pakistan that has tried to explore the impact and predictors of delay and TB treatment results in IDPs. Therefore, the findings of our study might be utilized as standard information for upcoming studies. The study furthermore highlighted the need for intervention in TB prevention and control strategies, especially among IDPs, to provide timely TB related care after displacement. Care-seeking behavior and delays in TB diagnosis in Pakistani IDPs will likely be further trampled by the beginning of the COVID-19 pandemic. This statement is supported by a WHO report that stated TB care could cause an additional half a million TB deaths [65].

## 5. Conclusions

A significant challenge for TB control is delayed case identification. This delay in TB diagnosis and treatment initiation should be reduced to the least possible in the case of human displacement, as migrants and internally displaced persons are more likely to experience interrupted health care because of their overall exclusion from social and public health services. Therefore, it is imperative to understand the barriers to providing public health care services, particularly in preventing and treating TB. Community engagement and multisectoral collaboration can improve the delivery of critical health services during displacement. Early screening tests are essential for infection control and treatment in a displacement situation. Empowering the patient by increasing the patient-centred approach involves the crisis/emergency affected TB patient in decision-making. The study further indicates that future TB control policies should address all issues of IDP TB patients in the country.

## Figures and Tables

**Table 1:** Sociodemographic characteristics of the TB patients enrolled in the study.

Variables	IDPs *n* (%)
Residence	
Village	194 (49.6)
City	59 (15.1)
Camp	138 (35.3)
Age (years)	
18–25	107 (27.4)
26–35	93 (23.8)
36–45	47 (12)
46–55	66 (16.9)
56–65	78 (19.9)
Gender	
Male	204 (52.2)
Female	187 (47.8)
Education	
Literate	57 (14.6)
Illiterate	334 (85.4)
Habitation density	
Low Density	66 (16.8)
High Density Overcrowded	159 (40.7) 166 (42.5)
Distance From Health Care Center	
≤5	32 (8.2)
6–15 km	92 (23.5)
16–30 km	133 (34)
>30 km	134 (34.3)
Health-seeking behavior	
Visited non-formal health provider	286 (73.1)
Visited a formal health provider	105 (26.9)
Self-Medication	
Yes	147 (37.6)
No	244 (62.4)
Category of treatment	
Category I	346 (88.5)
Category II	45 (11.5)
Have you ever heard of TB before diagnosis?	
Yes heard of TB	49 (12.5)
Not heard of TB	342 (87.5)
Number of TB patients at House	
Family members have TB	74 (18.9)
Family Member do not have TB	317 (81.1)
Perceived to be stigmatized	
Not stigmatized	112 (28.6)
Stigmatized	279 (71.4)

**Table 2:** TB Baseline signs and symptoms *n* = 391 (≤30 days vs. >30).

TB Baseline Signs and Symptoms	Total Number *n* (%)	Delay ≤ 30 Days	Delay > 30 Days	*p* Value
Cough	381 (97.4)	158 (41.5)	203 (58.5)	0.15
Chest pain	319 (81.6)	116 (36.4)	203 (63.6)	<0.001
Fever	311 (79.5)	98 (31.5)	213 (68.5)	<0.001
Loss of appetite	345 (88.2)	154 (39.8)	219 (60.2)	0.08
Night sweats	314 (80.3)	122 (38.9)	192 (61.2)	0.06
Bodyweight loss	304 (77.7)	108 (35.5)	194 (64.5)	<0.001

Note: Depends upon on patients’ response, more than one symptom, measured among patients who stated productive cough, *p* ≤ 0.05.

**Table 3:** Logistic Regression analysis of factors associated with prolonged delay in treatment IDPs Predicted probability is >30.

Factors	Delay ≤ than 30 Days	Delay > than 30 Days	Crude OR (95%-CI)	Adjusted OR (95% CI)
Residence				
Village	80 (41.2)	114 (58.8)	Reference	Reference
City	47 (79.7)	12 (20.3)	0.34 (0.27–0.93)	0.42 (0.16–1.18)
Camp	33 (23.9)	105 (76.1)	1.01 (0.48–2.11) *	1.39 (0.55–2.34)
Age (years)				
15–25	65 (60.7)	42 (39.3)	Reference	Reference
26–35	42 (45.2)	51 (54.8)	1.87 (1.07–3.30)	2.17 (0.84–5.34)
36–45	17 (36.2)	30 (63.8)	2.73 (1.34–5.55)	1.13 (0.40–3.27)
46–55	15 (22.7)	51 (77.3)	5.26 (2.62–10.3) *	3.37 (1.35–9.37) *
56–65	21 (26.9)	57 (73.1)	4.20 (2.23–7.91) *	2.66 (1.00–7.07) *
Gender				
Male	110 (53.9)	94 (46.1)	Reference	Reference
Female	50 (26.7)	137 (73.7)	3.20 (2.09–4.90) *	2.42 (1.21–4.81) *
Education				
Literate	24 (42.1)	33 (57.9)	Reference	Not included
Illiterate	136 (40.7)	198 (59.3)	1.05 (0.59–1.87)	
Place of living density				
Low Density	34 (51.5)	32 (48.5)	Reference	Not included
High Density	63 (39.6)	96 (60.4)	1.61 (0.90–2.88)	
Overcrowded	63 (38)	103 (62)	1.73 (0.97–3.08)	
Distance From Health Care Center				
≤5	26 (81.2)	6 (18.8)	Reference	Reference
6–15 km	49 (53.3)	43 (46.7)	3.80 (1.43–10.1)	1.77 (0.72–12.8)
16–30 km	62 (46.6)	71 (53.4)	4.96 (1.91–12.8)	2.13 (0.58–9.25)
>30 km	23 (17.2)	111 (82.8)	20.9 (7.73–56.5) *	4.13 (1.02–16.6) *
Health-seeking behavior				
Visited formal health provider	87 (82.9)	18 (17.1)	Reference	Reference
Visited non-formal health provider	73 (25.5)	213 (74.5)	14.1 (7.95–25.01) *	8.81 (1.37–19.46) **
Self-medication				
Yes	27 (18.4)	120 (81.6)	Reference	Reference
No	133 (54.5)	111 (45.5)	5.32 (3.27–8.67) *	2.72 (1.37–5.37) *
Category of treatment				
Category I Category II	148 (42.8) 12 (26.7)	198 (57.2) 33 (73.3)	Reference 2.05 (1.02–4.11) *	Reference 1.88 (0.68–5.18)
Have you ever heard of TB before diagnosis				
Yes heard	44 (89.2)	5 (10.2)	Reference	Reference
Not heard of	116 (33.9)	226 (66.1)	17.1 (6.61–44.0) *	11.39 (3.31–39.14) **
Number of TB patients at House				
Family members have TB	138 (22.5)	179 (77.5)	Reference	Reference
Family members do not have TB	22 (29.7)	52 (70.3)	1.82 (1.05–3.14) *	1.94 (0.78–4.79)
Perceived to be stigmatized				
Not stigmatized	95 (84.8)	17 (15.2)	Reference	Reference
Stigmatized	65 (23.3)	214 (76.7)	18.3 (10.2–33) *	8.81 (3.99–19.4) **

OR (odds ratio), CI (confidence interval), (Univariate analysis *p* < 0.15 is considered significant), Multivariate model was significant, with chi square = 270.6 (Df 16, *n* = 391), *p* < 0.005, Hosmer-Lemeshow statistic chi square = 6.65 (Df = 8, *n* = 391), *p* > 0.05, Collinearity (Variance inflation factor = 10), Tolerance value < 0.1, Reference category (more than 30 days), * *p* ≤ 0.05; ** *p* ≤ 0.01.

**Table 4:** Predictors associated with treatment outcomes in internally displaced TB patients.

Variables	IDPs *n* (%)	USO *n* (%)	Crude OR (95%-CI)	Adjusted OR (95%-CI)
Delay diagnosis				
Less than 30 days	160 (39.9)	13 (18.8)	Reference	Reference
More than 30 days	231 (57.6)	56 (81.2)	3.61 (1.94–6.8) *	2.60 (1.06–6.40) *
Residency				
Village	194 (49.6)	29 (42.1)	Reference	Reference
City	59 (15.1)	7 (10.1)	0.76 (0.31–1.85)	1.50 (0.55–4.08)
Camp	138 (35.3)	33 (47.8)	1.78 (1.02–3.11) *	1.41 (0.76–2.64)
Gender				
Male	204 (52.7)	31 (44.9)	Reference	Not included
Female	187 (47.8)	38 (55.1)	1.42 (0.84–2.40)	
Age (years)				
15–25	107 (27.4)	10 (14.5)	Reference	Reference
26–35	93 (23.8)	10 (14.5)	1.16 (0.46–0.29)	0.97 (0.36–2.57)
36–45	47 (12)	7 (10.10)	1.69 (0.60–4.77)	1.26 (0.42–3.76)
46–55	66 (16.9)	20 (29.0)	4.21 (1.82–9.73) *	3.15 (1.27–7.80) *
56–65	78 (19.9)	22 (31.9)	3.81 (1.68–8.62) *	3.00 (1.25–7.19) *
Education				
Literate	57 (14.6)	33 (47.8)	Reference	Not Included
Illiterate	334 (85.4)	36 (52.1)	1.00 (0.48–2.11)	
Number of Room/Tents Per house				
Low Density	66 (16.9)	11 (15.9)	Reference	Not Included
High Density	159 (40.7)	22 (31.9)	0.80 (0.36–1.76)	
Overcrowded	166 (42.5)	36 (52.2)	1.38 (0.65–2.91)	
Distance From Health Care Center				
≤5	32 (8.2)	4 (5.8)	Reference	Not Included
6–15 km	92 (23.5)	14 (20.3)	1.25 (0.38–4.13)	
16–30 km	133 (34)	17 (24.6)	1.02 (0.32–3.28)	
>30 km	134 (34.3)	34 (49.3)	2.38 (0.77–7.27)	
Health-seeking behavior				
Visited a formal health provider	105 (26.9)	10 (14.5)	Reference	Reference
Visited non-formal health provider	286 (73.1)	59 (85.5)	1.72 (0.89–3.30) *	1.43 (0.60–3.35)
Self-medication				
Yes	147 (37.6)	37 (53.6)	Reference	Reference
No	244 (62.4)	32 (46.4)	1.79 (1.06–3.03) *	1.42 (0.77–2.62)
Have you ever heard of TB before diagnosis?				
Yes heard	49 (12.5)	6 (8.7)	Reference	Reference
Not heard of	342 (87.5)	63 (91.3)	1.61 (0.66–3.98) *	1.40 (0.49–4.008)
Number of TB patients at House				
Family members have TB	74 (18.9)	20 (28.9)	Reference	Not Included
Family members do not have TB	317 (81.1)	49 (71.8)	0.80 (0.42–1.52)	
Category of treatment				
Category I	346 (88.5)	49 (71)	Reference	Reference
Category II	45 (11.5)	20 (29)	4.84 (2.50–9.39) *	4.80 (1.99–8.34) *
Perceived to be stigmatized				
Not stigmatized	112 (28.6)	12 (17.4)	Reference	Not Included
Stigmatized	279 (71.4)	57 (82.6)	2.14 (1.10–4.16)	

USO (unsuccessful outcomes), OR (odds ratio), CI (confidence interval), (Univariate analysis *p* < 0.15 is considered significant), Multivariate model was significant, with chi square = 49.34 (Df 11, *n* = 391), *p* < 0.005, Hosmer-Lemeshow statistic chi square = 7.88 (Df = 8, *n* = 391), *p* > 0.05, Collinearity (Variance inflation factor = 10), Tolerance value *p* < 0.1, Reference category (Unsuccesful outcomes), * *p* ≤ 0.05.

## Data Availability

The data sets used and analyzed during the current study are available from the corresponding author on reasonable request.
